# Willingness of Participation in an Application-Based Digital Data Collection among Different Social Groups and Smartphone User Clusters

**DOI:** 10.3390/s23094571

**Published:** 2023-05-08

**Authors:** Ákos Máté, Zsófia Rakovics, Szilvia Rudas, Levente Wallis, Bence Ságvári, Ákos Huszár, Júlia Koltai

**Affiliations:** 1 MTA–TK Lendület “Momentum” Digital Social Science Research Group for Social Stratification, Centre for Social Sciences, Tóth Kálmán Utca 4, 1097 Budapest, Hungary; 2 Institute for Political Science, Centre for Social Sciences, Tóth Kálmán Utca 4, 1097 Budapest, Hungary; 3 Department of Social Research Methodology, Institute of Empirical Studies, Faculty of Social Sciences, Eötvös Loránd University, Pázmány Péter sétány 1/A, 1117 Budapest, Hungary; 4 Computational Social Science-Research Center for Educational and Network Studies (CSS-RECENS), Centre for Social Sciences, Tóth Kálmán Utca 4, 1097 Budapest, Hungary; 5 Department of Sociology, Institute of Social and Political Sciences, Corvinus University of Budapest, Fővám tér 8, 1093 Budapest, Hungary; 6 Institute for Sociology, Centre for Social Sciences, Tóth Kálmán Utca 4, 1097 Budapest, Hungary

**Keywords:** data collection methods, survey experiment, smartphone data, digital trace data, willingness of participation

## Abstract

The main question of this paper is what factors influence willingness to participate in a smartphone-application-based data collection where participants both fill out a questionnaire and let the app collect data on their smartphone usage. Passive digital data collection is becoming more common, but it is still a new form of data collection. Due to the novelty factor, it is important to investigate how willingness to participate in such studies is influenced by both socio-economic variables and smartphone usage behaviour. We estimate multilevel models based on a survey experiment with vignettes for different characteristics of data collection (e.g., different incentives, duration of the study). Our results show that of the socio-demographic variables, age has the largest influence, with younger age groups having a higher willingness to participate than older ones. Smartphone use also has an impact on participation. Advanced users are more likely to participate, while users who only use the basic functions of their device are less likely to participate than those who use it mainly for social media. Finally, the explorative analysis with interaction terms between levels has shown that the circumstances of data collection matter differently for different social groups. These findings provide important clues on how to fine-tune circumstances to improve participation rates in this novel passive digital data collection.

## 1. Introduction

Empirical social science, especially survey research, is increasingly challenged by growing non-response rates. The systematic exclusion of certain social groups calls into question the representativeness of the samples used and the accuracy of the estimates derived from them [1,2]. It is therefore essential to investigate the potential selection biases caused by non-response to units. This is especially true for innovative data collection techniques that go beyond traditional survey methods by using new instruments to collect new types of data [3,4,5].

The vast majority of empirical studies in the social sciences still rely on traditional, survey-based data collection procedures. However, a growing number of experiments are taking advantage of the opportunities offered by the information revolution and the development of infocommunication technologies, and are developing new procedures and methods for data collection [6,7,8,9]. The vast majority of empirical studies in the social sciences still rely on traditional, survey-based data collection procedures. However, a growing number of experiments are taking advantage of the opportunities offered by the information revolution and the development of infocommunication technologies and are developing new procedures and methods for data collection [6,7,8,9]. In this paper we join these efforts and present the results of an exploratory study conducted in Hungary. This involved an online survey to determine willingness to participate in a future research project based on smartphone data collection.

In our study, we seek answers to three questions: first, how the willingness to participate in smartphone-based data collection is related to the socio-demographic characteristics of the individuals; second, how participation is influenced by the different types of smartphone use; and third, to what extent can the selection biases caused by the previous two factors be reduced by careful planning of the survey design.

### Factors Determining Willingness to Participate

The development and application of innovative data collection techniques is necessitated by the fact that traditional survey methods are encountering increasing difficulties as non-response rates rise [1,2,10]. However, with novel and innovative techniques—such as online data collection, data donation or the use of smartphone applications—it may be possible to reach social groups (e.g., young people) who are difficult to persuade to participate in social science research using conventional survey methods [3,4,5,8,11,12]. In addition to reaching potentially new groups of respondents, however, a far greater advantage of these new techniques is that we can measure digital activity with a level of accuracy and detail that is not possible with traditional surveys.

According to the method of data collection, a distinction can be made between active and passive data collection [5,13]. Traditional survey-based techniques almost exclusively use the active form of data collection. Data is collected in such a way that respondents actively answer questions formulated in advance by the researcher. In this case, the researcher assumes, or is forced to assume, that respondents understand the questions in the survey and have sufficient information to answer them. Another important assumption is that respondents are willing to answer the questions honestly and to the best of their knowledge and belief [14,15,16]. It is clear that these conditions, or some of them, are regularly not fulfilled and the researcher has limited possibilities to verify them.

Passive data collection, on the other hand, does not require the data provider to actively participate in the collection of the information. Smartphone-based data collection techniques, for example, obtain information through an application that accesses data generated by sensors built into the device or by the smartphone’s operating system. This can include information about people’s online activities, app usage, location data or even their health status [9,13,17,18,19,20]. A key advantage of passive data collection is that it avoids many of the pitfalls of active techniques, as it does not rely on the active role of participants in data collection. This can lead to more accurate measures of digital activity.

However, it is also clear that while innovative data collection techniques have many advantages over traditional survey-based methods, there are also some problems that pose a major challenge to the widespread use of these techniques.

All social science data collection is based on a strong relationship of trust between researchers and data providers [21,22,23]. If this relationship of trust does not exist, members of society will refuse to participate in research. Refusal to participate is a growing problem in traditional data collection, but it is even more pronounced in passive data collection (including smartphone-based techniques), which require access to particularly sensitive personal data when they aim to observe people’s digital behaviour. It is no coincidence that willingness to participate in this type of data collection is generally lower than in traditional surveys [13,24,25,26,27,28,29]. Depending on the research design and specific data collection, actual and hypothetical willingness to participate varies widely. (For a comparative overview see [5,12])

Digital footprints can be used for research in many ways. The constantly evolving technological environment puts these data collections under constant pressure to innovate. On desktops, the most common methods are customised browsers or browser plug-ins that log website traffic and methods that use screen scraping. On mobile devices, it is possible to monitor a wide range of user activity by directly accessing sensors and local databases via APIs. The third method is data donation, where users donate their personal data generated and stored by social media services, e-commerce, etc. Research has looked at a variety of factors that influence willingness to participate and the level of collaboration. The type of organisation conducting the research (i.e., academic research is favoured over commercial research) and the purpose of the research (to what extent it serves the public good) seem to have an impact on willingness to participate [30,31]. Empirical experience to date is contradictory in terms of preference for full, partial or no transparency of the actual data users provide from their device when participating [21,32]. However, the sensitivity and personal relevance of the data is perceived to be the most decisive factor [31,32,33]. So far, research on the role of incentives has been rather hypothetical, i.e., surveys have been conducted to measure willingness to participate in the case of different incentives. Experience so far shows that there are no easy-to-apply formulas for the amounts of incentives for which people are willing to participate in passive data collection or to donate their data. Higher amounts are associated with higher willingness to participate, but beyond a certain point, higher payments no longer improve the level of engagement [12,21,30,34]. Compared to previous studies, we examine these factors in a controlled survey experiment that allows us to test whether the different factors in the research design affect willingness to participate differently in different social groups.

Since data collection via smartphone directly observes the digital behaviour of individuals, the question of research ethics arises to a much greater extent. Therefore, data collection needs to be much more carefully and thoroughly planned to avoid data containing personal information and to ensure proper anonymisation of sensitive data. In addition, the safe storage of particularly sensitive data must be ensured [9,35,36].

Due to the higher refusal rate, smartphone-based data collection techniques may also lead to difficulties in reaching certain social groups. For example, it is obvious that a prerequisite for participation in a smartphone-based data collection is that the respondent owns such a device. In Hungary, more than 90% of the population owns a smartphone, but those who are excluded due to the lack of smart devices still cause systematic selection bias. In fact, the most disadvantaged and vulnerable populations are systematically excluded from data collection. Participation in this type of innovative data collection also requires a minimum level of digital literacy, which automatically limits the reach of certain social groups, e.g., the digitally excluded groups [37,38,39,40]. However, this innovative method of data collection can reach previously less accessible groups (e.g., the younger generation) at a higher rate. In our exploratory study, we focus on these issues by investigating which social groups can be reached with passive digital data collection and which would refuse to participate in such a survey. We investigate the role socio-economic variables play in willingness to participate. In addition, we use a survey experiment to estimate how certain elements of the research design affect the willingness to participate of different social groups and different types of smartphone users.

Based on the literature, we explore three questions related to participation rates and research design.

First, previous studies show that participation in traditional surveys is systematically related to demographic and socio-economic characteristics of respondents. In Hungary, for example, non-response is systematically higher among young people, people living in the capital and people with a more favourable social position [2]. However, we expect socio-economic factors to have a different effect in the case of innovative, sensor-based data collection. For example, we expect that, similar to other studies, younger people are more likely to participate in smartphone-based data collections [5,27]. Our first question is therefore: How is the willingness to participate in a smartphone-based data collection related to the demographic and socio-economic characteristics of the respondents?

Second, smartphone ownership and the nature of smartphone use itself is related to individuals’ socio-economic characteristics [32,34]. Based on these findings, we hypothesise that the type of smartphone use itself might also be related to participation in data collection. In our work, we create a typology of smartphone users based on intensity and different types of device use and explore how this relates to willingness to participate. Based on previous work, we hypothesise that people who use their smartphone more intensively, diversely and progressively are more willing to participate in data collection [27,38]. Therefore, our second question is whether and how smartphone use is related to willingness to participate.

The third main question of the article is to what extent willingness to participate in a sensor-based data collection depends on the research design itself. Similar to [5], but with a different measurement of the dependent variable, we investigate how the survey can be designed to minimise non-response and encourage participation. In this context, we examine the role of various factors (survey organiser, amount of data collected, duration of the survey, level of incentives offered to respondents, interruption and control of data collection) that can have a major impact on willingness to participate in data collection [5].

However, we would like to explore not only how these factors relate to participation, but also how they work across different social groups and smartphone user groups. This part of the analysis is mainly exploratory, although the basis of these analyses is our assumption that incentives, duration of data collection or other factors do not have the same effect on willingness to participate among higher and lower status individuals or among basic and advanced smartphone users. The strength and direction of these effects are difficult to predict because people from different social backgrounds and with different ways of using their phones may have different sensitivities to elements of the research design, resulting in different participation rates. For this reason, we systematically investigate (i) how the interactions between social status and different factors of the research design and (ii) how the interactions between types of phone users and factors of the research design relate to willingness to participate in sensor-based data collection. Our fourth question is therefore: What are the effects of the different elements of the research design on the different social groups and types of smartphone users studied and how do they affect willingness to participate?

## 2. Materials and Methods

**Figure 1:**
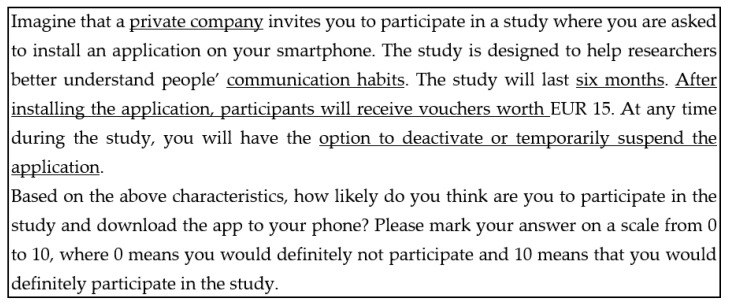
An example of a vignette. Underlined features of the situation were systematically varied on the vignettes presented to the respondents.

In 2021, an online data survey was conducted among Hungarian internet users. The sample was selected from the online panel of a market research company and is representative of Hungarian internet users by gender, age, highest level of education, type of establishment and region. The sample size was 1000. The questionnaire included questions about the socio-demographic characteristics of the respondents, their attitudes towards various aspects of the digital world and their use of information and communication technology (ICT) devices. In addition to these questions, we also created a special block with a factorial survey design. In this block, we asked respondents about different situations, which we called ‘vignettes’. Each vignette contained a hypothetical scenario about a smartphone-based data collection, and respondents had to indicate how likely they would be to participate in such research [5]. An example of the vignettes is shown in Figure 1. We systematically varied five different features of the situation (underlined in Figure 1).

The different characteristics were the following: organisation of the study (decision-maker; a private company; a scientific research institute), data collected (spatial movement; mobile phone use; communication habits; spatial movement and mobile phone use; spatial movement and communication habits; mobile phone use and communication habits; all three), duration of the study (one month; six months), incentive (EUR 15 (HUF 5000) after installation of the application; EUR 15 (HUF 5000) after completion of the study; EUR 15 (HUF 5000) after installation of the application; and EUR 15 (HUF 5000) after completion of the study), interruption and control (the user cannot interrupt data collection; the user can temporarily interrupt data collection; the user can temporarily interrupt data collection and review the data and approve its transfer). Following Jasso’s [41] research design strategy, we randomly selected 150 out of the 378 different combinations of these dimensions—i.e., out of all possible situations—and divided them into 15 different packages (called decks) with 10 situations each. Each deck was randomly assigned to a respondent. One respondent rated one deck, i.e., ten different situations. In total, the 1000 respondents rated 10,000 vignettes. This method combines the advantages of surveys and experiments: Due to the large sample size, not only can the main effects of the stimuli be estimated, but also their interaction with each other and with the respondents’ characteristics.

Of the socio-demographic characteristics of the respondents, we used gender, age, type of settlement and a typology of activity and education as control variables. Gender was measured as a binary variable (men and women). Age and type of settlement were measured in three categories (18–39, 40–59, 60+ years old; and correspondingly capital, city, village). The typology comprised eight categories: employed (including employers and entrepreneurs), unemployed, retired and other inactive—all divided into skilled (tertiary) and unskilled (maximum secondary) categories based on education level. Gender, age and settlement type were used as control variables and the analyses focused on the role of typology in the decision-making process about participation.

In addition to socio-demographic characteristics, we also included a variable on smartphone use in the models. Based on (1) 15 different activities listed in the questionnaire for which one can use a smartphone, (2) the average self-estimated daily duration of smartphone screen use, and (3) self-reported smartphone use skills (see original items in Appendix A, Table A2), we defined 5 clusters using latent class analysis [42] using R’s poLCA package (v1.6.0.1) [43]. The main clustering factors were social media use, time spent on general entertainment and use of some specific device features (i.e., camera and related apps). The names and short descriptions of these clusters are as follows. For *social media and entertainment users*, smartphone use essentially revolves around social media and entertainment. They tend to be younger, have average (self-assessed) technical knowledge and spend an above-average amount of time on the smartphone. *Non-social-media users* are less focused on social media and entertainment in general or rarely perform these activities on their mobile devices. Young people are underrepresented in this group, otherwise they show an average level of technical knowledge and screen time. *General basic users* do not consider their smartphone as a universal tool, but use it mainly for making phone calls, searching for basic information and doing everyday tasks. They are characterised by a low level of technical knowledge. The main characteristic of *camera users* is the prominent importance of the device as a camera, complemented by the use of apps to create media content. *Advanced users* are the jacks-of-all-trades who use their smartphone in a variety of ways. They have a secure technological knowledge and spend an above-average amount of time with their phone. The descriptive statistics for these variables are summarised in Table 1.

When we examine the five groups of smartphone users in terms of age, education and position in the labour market (see Table 2), we find that the results are consistent with the literature [38,44]. Advanced users are generally younger (only 7.7% of respondents aged 60 and older belong to this group), while social media use is more consistent across the sample. Smartphone use is less clear when looking at education and labour market activity. While 34% of the skilled and employed part of the sample are advanced users, this percentage is 34.8% for the unskilled–other category. The main users of the camera are the unskilled–retired (27.8%), while the unskilled–employed are among the groups least likely to use their device with the camera only.

**Figure 2:**
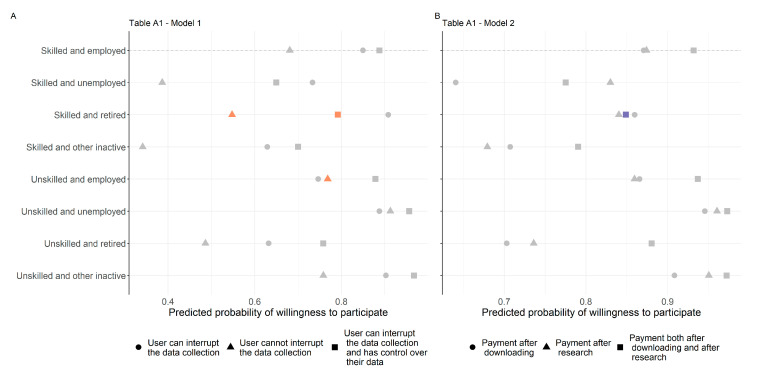
Predicted values of cross-level interactions, Table A1, Models 1 and 2. (**A**) Respondent level socio-demographic variables and interruption and control over the data collection. (**B**) Respondent level socio-demographic variables and incentives. Note: Significant interactions are highlighted with colours, nonsignificant ones are grey.

**Figure 3:**
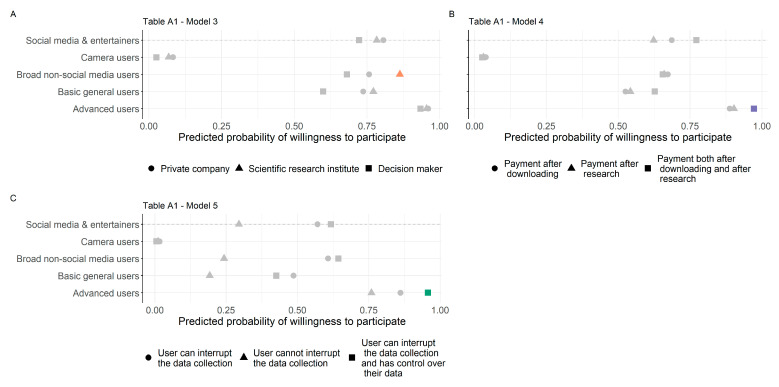
Predicted values of cross-level interactions, Table A1, Models 3–5. (**A**) Respondents’ smartphone usage and organiser of the research. (**B**) Respondents’ smartphone usage and incentives. (**C**) Respondents’ smartphone usage and interruption and control over the data collection. Note: Significant interactions are highlighted with colours, nonsignificant ones are grey.

We analysed the data with multilevel models [45,46], using the binary version of willingness to participate as the dependent variable, recoding it from 0 to 5 into ‘rather not participate’ and from 6 to 10 into ‘rather participate’. We built hierarchical models using R’s lme4 package (v1.1-31) in the following way [47]. We included only the independent variables at the vignette level in the first model (Table 3, Model 1), then respondent characteristics in the second model (Table 3, Model 2), and smartphone user groups in the third model (Table 3, Model 3). We then successively tested the interactions between respondents’ socio-demographic characteristics and vignette dimensions (e.g., the interaction between the incentive and the education and activity typology) and then the interactions of smartphone user groups with different vignette-level variables (e.g., the interaction between the research organiser and user groups) on willingness to participate (Figure 2 and Figure 3—see detailed results in Appendix A, Table A1). The interaction plots were made with the ggeffects (v1.1.4) R package [48]. To assess model fit, we used the Akaike information criterion (AIC) and the Bayesian information criterion (BIC), both of which provide information about model fit while penalising the inclusion of additional variables in the model. The lower the AIC and BIC values, the better the model.

## 3. Results

The results of the models focusing on the socio-demographic characteristics and smartphone use behaviour of the respondents are presented in Table 3. In the first model, we tested the effects of the variables at the vignette level. The results of the base model (Model 1 in Table 3) show that respondents are less willing to participate in a survey conducted by policymakers than by private companies. Respondents are also less willing to participate in a study if the duration of data collection is longer, preferring a period of one month. Compared to respondents who can interrupt data collection, they are less willing to participate in a survey if they have no control over interrupting the process. They are more likely to participate if it is possible for users to temporarily pause the data collection, review the data and authorise its sharing. The study of the effects of the vignette variables has shown that the level of incentives matters. Compared to a study without incentives, respondents are more likely to participate if they receive EUR 15 after installing the application and EUR 15 after completing the study. These effects in Model 1 (in Table 3) are also robust to different model specifications, such as the introduction of socio-economic variables and cross-level interactions.

By introducing additional variables into the model (see Model 2 in Table 3), we were able to determine the impact of socio-demographic variables on willingness to participate by controlling for respondents’ gender, age, settlement type, education level and active/inactive economic status (see the eight-category typology of the latter two). Compared to the youngest age group (18–39 years), willingness to participate in data collection was lower among both middle-aged (40–59 years) and elderly (60+ years) respondents. Examination of the gender of the participants revealed that women were less likely to participate in research to capture digital traces of their everyday life compared to men.

In examining the typology based on participants’ smartphone use, we noted the differences between levels of user engagement and activities (see Model 3 in Table 3). According to the AIC and BIC scores, this model fits the data better. Compared to those who typically use their smartphone for social media and entertainment options, those who only use the camera and basic options are less likely to be engaged. However, those we can call advanced users (i.e., those who use almost all the features a smartphone can offer) declared a higher willingness to participate.

We also examined the interactions of the different variables at the vignette level with the constructed eight-category typology (see Table A1 in Appendix A for details) to gain insight into the mixed effects that variables can have on interest in participation. The results are shown in Figure 2A,B. Compared to the educated, active group of respondents, the qualified and retired respondents are less concerned about control over data collection when deciding whether to participate, but the lack of interruption is a red flag and in this circumstance they would participate even less than the reference group. However, compared to the same reference group, the less educated active workers, the lack of interruption is less important when they declare their willingness to participate. Regarding the role of the incentive, we can see that those who are educated and retired rely less on the level of the incentive in their decision than those who are also educated but active.

Based on the interactions between smartphone use and vignette-level variables, we can observe several cross-effects (see Figure 3 and Models 3–5 in Table A1).

For those who belong to the broad category of non-social-media users (i.e., those who use several features of their smartphone but not social media), it is more likely to be a positive factor if the organiser of the data collection is a research institute rather than a private company than for those who mainly use social media and entertainment (Model 3 in Table A1, visualised in Figure 3A). Compared to the same reference group, the level of incentive is more important for advanced users, so they are more likely to participate if they are offered EUR 15 after installing the app and EUR 15 after completing the study (Model 4 in Table A1, visualised in Figure 3B). Control over data collection is also more important to them when deciding to participate; advanced users are more likely to participate if they have the option to temporarily pause data collection, review the data and approve its transfer compared to those who focus on social media and entertainment options (Table A1, Model 5, visualised in Figure 3C).

## 4. Discussion

In our study, we investigated how willingness to participate in smartphone-based data collection is related to individuals’ socio-demographic characteristics and type of smartphone use. Our first and second research questions focused on the influence of socio-economic variables and smartphone use on respondents’ willingness to participate in passive data collection. We find that both socio-demographic characteristics and smartphone use influence the outcome, albeit to different degrees. Our results show that of the socio-demographic variables, age has the greatest influence, with younger age groups having a higher willingness to participate than older age groups. Interestingly, education and labour market status do not have a significant impact on willingness to participate, even when smartphone use behaviour is excluded.

We also find that the more smartphone features respondents use, the greater their willingness to participate in passive data collection. Comparing the different smartphone user groups with those who use the smartphone only for social media and entertainment purposes, willingness to participate is lower among camera users. On the other hand, the willingness to participate is significantly higher among advanced users. Our results are in line with previous studies [21,27,49]. They also show that digital literacy is a crucial factor to consider when designing studies that rely on passive data collection.

To answer our third research question, how design choice affects participation outcomes, we used vignette-level data from the survey experiment. In line with [5], we found that the type of data collected did not significantly affect willingness to participate. However, the ability to interrupt and/or control the survey, a greater incentive and a shorter duration of data collection increased willingness to participate. Among respondents, interruption was considered more important than control over their data. Advanced users valued the incentives they received both after downloading the app and at the end of the study, as well as the ability to pause the survey and have control over their data, compared to the social media and entertainment group. The duration of data collection of six months compared to one month significantly reduced willingness to participate.

Exploratory analysis with the interaction terms shows that the circumstances of data collection matter differently for different social groups. For those who are educated and retired, both control over data collection and the level of incentive are less important than for those who are also educated but still active. Interestingly, the dual incentive does not play as big a role for the educated retirees in their decision to participate as it does for the educated active. We also found that the organiser of the research, the level of the incentive and the control over the data play different roles for different groups of smartphone users.

Nevertheless, our study is subject to several limitations. First, the situations assessed by the respondents are hypothetical, so their real-life activities may be different. Since installing the application or verifying the data is an energy- and time-consuming process (which also depends on the digital skills of the participant), we can assume that such a requirement may discourage participation. The actual willingness to participate may also be influenced by recent scandals related to the misuse of personal data, as in the case of Cambridge Analytica or Pegasus. Therefore, these results are suitable for studying trends between different social groups (which is what we used them for), but less so for estimating actual participation rates. Another line of research that solves this limitation could be the comparison of the evaluation of such hypothetical situations with real behaviour, as in the case of [23]. Another limitation relates to the generalisability of the results. Our study focuses on a single country, namely Hungary. However, we can assume that a variety of factors that vary between societies and cultures may influence participation in innovative data collection. For example, in the case of data donation, Kmetty et al. [50] found that circumstances of the research affect potential participants’ decision differently in Hungary and in the US. However, as we have argued above, our results seem to be comparable to those of other countries, suggesting that there are overarching patterns that can be observed in different cultures and countries.

Our results show that the circumstances of the survey affect participation rates differently across social groups. Both socio-demographic and digitisation-related factors (such as smartphone use) play a role. These findings can be useful for researchers in designing data collection when the inclusion of certain social groups is particularly important. In order to minimise the exclusion of certain social groups on which the research focuses, it is worthwhile to design such a data collection in a way that optimises the willingness of these groups to participate. The results may also be useful for researchers targeting a more general population, as they can be more aware of the potential selection bias in their sample.

## Figures and Tables

**Table 1:** Descriptive statistics of socio-economic variables. All numbers are in percentage and the proportions are computed with sample weights.

**Age**							
18–39			40–59		60+		
42.9			39.4		17.7		
**Gender**							
		Male			Female		
		48.2			51.8		
**Settlement**							
Capital			City		Village		
21.2			52.1		26.7		
**Education and Labour Market Activity**							
Skilled and employed	Skilled and unemployed	Skilled and retired	Skilled and other	Unskilled and employed	Unskilled and unemployed	Unskilled and retired	Unskilled and other
42.9	3.4	9.5	8.6	20.2	3.5	6.5	5.4
**Smartphone User Clusters**							
Social media and entertainment users		Broad non-social-media users		Basic general users		Camera users	Advanced users
31.2		12.0		18.7		9.1	29.0
**Willingness to Participate**							
No				Yes			
47.2				52.8			

**Table 2:** The relationship between smartphone user clusters and socio-economic variables. Row percentages.

	Smartphone User Cluster				
Age	Social Media and Entertainment Users	Broad Non-Social-Media Users	Basic General Users	Camera Users	Advanced Users
18–39	34.4	8.0	8.0	2.0	47.5
40–59	32.6	14.3	21.6	9.1	22.3
60+	21.9	14.8	32.9	22.6	7.7
	**Smartphone User Cluster**				
**Education and Labour Market Activity**	**Social Media and Entertainment Users**	**Broad Non-Social-Media Users**	**Basic General Users**	**Camera Users**	**Advanced Users**
Skilled and employed	30.3	13.8	15.5	6.3	34.0
Skilled and unemployed	14.3	33.3	23.8	9.5	19.0
Skilled and retired	21.9	15.6	33.3	19.8	9.4
Skilled and other inactive	41.2	2.5	6.2	0.0	50.0
Unskilled and employed	40.4	9.1	18.2	11.1	21.2
Unskilled and unemployed	46.7	13.3	13.3	0.0	26.7
Unskilled and retired	19.4	2.8	47.2	27.8	2.8
Unskilled and other inactive	34.8	4.3	13.0	13.0	34.8

**Table 3:** Results of the multilevel models on the willingness to participate including vignette dimensions, socio-demographic variables and smartphone user typology. Unstandardized coefficients (* *p* < 0.05, ** *p* < 0.01, *** *p* < 0.001); standard errors of the coefficients are in brackets.

	DV: Willingness to Participate		
	Model 1	Model 2	Model 3
**Vignette-level variables**			
*Organiser of the research (ref: private company)*			
Scientific research institute	0.07 (−0.09)	0.07 (−0.1)	0.02 (−0.12)
Decision-maker	−0.39 *** (−0.09)	−0.39 *** (−0.1)	−0.52 *** (−0.11)
*Data collected (ref: spatial movement)*			
Mobile usage	−0.19 (−0.14)	−0.19 (−0.16)	−0.11 (−0.18)
Communication habits	−0.26 (−0.14)	−0.27 (−0.16)	−0.21 (−0.19)
Movement and usage	−0.19 (−0.13)	−0.19 (−0.15)	−0.12 (−0.17)
Movement and comm. habits	0.08 (−0.15)	0.08 (−0.17)	0.15 (−0.19)
Mobile usage and comm. habits	−0.08 (−0.14)	−0.08 (−0.16)	0.04 (−0.18)
Movement, usage and comm. habits	−0.07 (−0.14)	−0.07 (−0.16)	−0.09 (−0.19)
*Length of the research (Ref: one month)*			
Six-month duration	−1.15 *** (−0.07)	−1.16 *** (−0.08)	−1.31 *** (−0.1)
*Incentive (ref: after downloading the app)*			
After the end of the research EUR 15	0.08 (−0.09)	0.08 (−0.1)	0.01 (−0.12)
Both after downloading the app and at the end of the research EUR 15-EUR 15	0.61 *** (−0.09)	0.61 *** (−0.1)	0.63 *** (−0.12)
*Interruption and control (ref: user can interrupt the data collection)*			
User cannot interrupt the data collection	−0.98 *** (−0.09)	−0.99 *** (−0.1)	−1.08 *** (−0.12)
User can interrupt the data collection and has control over their data	0.20 * (−0.09)	0.2 (−0.1)	0.29 * (−0.12)
**Respondent level socio-demographic variables**			
Age 40–59 (ref: 18–39)		−1.52 *** (−0.46)	−0.44 (−0.56)
Age 60+		−2.76 *** (−0.76)	−0.77 (−0.89)
Gender (ref: men)		0.3321	−0.4 (−0.48)
Town (ref: capital)		0.85 (−0.51)	0.96 (−0.59)
Village		1.02 (−0.6)	0.97 (−0.69)
Skilled–retired (ref: skilled–employed)		−0.01 (−0.84)	−1.17 (−1.41)
Skilled–unemployed		−0.98 (−0.99)	−0.35 (−0.95)
Skilled–other inactive		−1.18 (−0.7)	−1.47 (−0.82)
Unskilled–employed		−0.05 (−0.6)	−0.18 (−0.73)
Unskilled–unemployed		1.14 (−1.33)	0.27 (−1.65)
Unskilled–retired		−0.63 (−1.06)	−0.5 (−1.25)
Unskilled–other inactive		0.71 (−1.07)	−0.57 (−1.35)
**Typology of smartphone use (ref: Social media and entertainment)**			
Broad non-social-media users			0.02 (−0.79)
Basic general users			−0.42 (−0.69)
Camera users			−3.69 *** (−0.94)
Advanced users			1.66 ** (−0.6)
AIC	6783.87	6773.36	5247.53
BIC	6892.03	6968.04	5463.43
Observations	10,000	10,000	7820
Groups (respondents)	1000	1000	782

## Data Availability

The data presented in this study are available on request from the corresponding author. The data are not publicly available due to contractual restrictions with the fieldwork agency conducting the online survey.

## Appendix A

**Table A1:** Results of the multilevel models on willingness to participate including vignette dimensions, socio-demographic variables, and smartphone user typology. Interactions between the respondents’ socio-demographic characteristics and the dimensions of the vignettes and the respondents’ smartphone usage and the dimensions of the vignettes. Unstandardized coefficients (* *p* < 0.05, ** *p* < 0.01, *** *p* < 0.001); standard errors of the coefficients are in brackets.

	DV: Willingness to Participate				
	Model 1	Model 2	Model 3	Model 4	Model 5
**Vignette-level variables**					
*Organiser of the research (ref: private company)*					
Scientific research institute	0.07 (−0.11)	0.08 (−0.1)	−0.14 (−0.22)	0.04 (−0.12)	−0.02 (−0.13)
Decision-maker	−0.42 *** (−0.11)	−0.41 *** (−0.1)	−0.47 (−0.24)	−0.55 *** (−0.12)	−0.58 *** (−0.12)
*Data collected (ref: spatial movement)*					
Mobile usage	−0.28 (−0.17)	−0.18 (−0.16)	−0.15 (−0.19)	−0.06 (−0.18)	−0.23 (−0.19)
Communication habits	−0.37 * (−0.18)	−0.26 (−0.17)	−0.2 (−0.2)	−0.19 (−0.19)	−0.36 (−0.2)
Movement and usage	−0.23 (−0.16)	−0.18 (−0.15)	−0.15 (−0.18)	−0.08 (−0.17)	−0.17 (−0.18)
Movement and comm. habits	0.04 (−0.18)	0.08 (−0.17)	0.15 (−0.2)	0.18 (−0.19)	0.07 (−0.21)
Mobile usage and comm. habits	−0.18 (−0.17)	−0.1 (−0.16)	0.03 (−0.19)	0.04 (−0.18)	−0.07 (−0.19)
Movement, usage and comm. habits	−0.1 (−0.18)	−0.07 (−0.17)	−0.12 (−0.2)	−0.09 (−0.19)	−0.1 (−0.21)
*Length of the research (Ref: one month)*					
Six-month duration	−1.29 *** (−0.09)	−1.19 *** (−0.09)	−1.39 *** (−0.1)	−1.33 *** (−0.1)	−1.48 *** (−0.11)
*Incentive (ref: after downloading the app)*					
After the end of the research EUR 15	0.09 (−0.11)	0.03 (−0.15)	−0.01 (−0.13)	−0.28 (−0.21)	−0.01 (−0.13)
Both after downloading the app and at the end of the research EUR 15-EUR15	0.68 *** (−0.11)	0.71 *** (−0.16)	0.66 *** (−0.13)	0.44 (−0.25)	0.73 *** (−0.13)
*Interruption and control (ref: user can interrupt the data collection)*					
User cannot interrupt the data collection	−0.98 *** (−0.16)	−1.01 *** (−0.11)	−1.13 *** (−0.13)	−1.09 *** (−0.12)	−1.16 *** (−0.23)
User can interrupt the data collection and has control over their data	0.33 (−0.18)	0.21 * (−0.1)	0.31 * (−0.12)	0.32 ** (−0.12)	0.19 (−0.31)
**Respondent level socio-demographic variables**					
Age 40–59 (ref: 18–39)	−1.42 ** (−0.49)	−1.37 ** (−0.48)	−0.89 (−0.62)	0.01 (−0.51)	−0.16 (−0.52)
Age 60+	−2.52 ** (−0.82)	1.9491	−0.83 (−0.9)	0.17 (−0.79)	−0.18 (−0.81)
Gender (ref: men)	−0.44 (−0.45)	−0.57 (−0.46)	−0.39 (−0.51)	−0.35 (−0.45)	0.22 (−0.45)
Town (ref: capital)	1.13 * (−0.52)	0.77 (−0.52)	0.77 (−0.63)	0.78 (−0.54)	1.12 * (−0.55)
Village	1.22 * (−0.6)	0.85 (−0.6)	0.67 (−0.75)	0.51 (−0.62)	1.11 (−0.63)
Skilled–retired (ref: skilled–employed)	0.56 (−0.92)	−0.1 (−0.86)	−2.15 (−1.49)	−0.43 (−1.26)	−0.3 (−1.43)
Skilled–unemployed	−0.72 (−1.08)	−1.33 (−0.98)	−0.68 (−0.98)	−0.02 (−0.83)	0.24 (−0.9)
Skilled–other inactive	−1.2 (−0.77)	−1.02 (−0.7)	−1.93 * (−0.84)	−0.53 (−0.77)	−1.29 (−0.82)
Unskilled–employed	−0.65(−0.67)	−0.04 (−0.59)	−0.37 (−0.79)	−0.64 (−0.66)	−0.45 (−0.79)
Unskilled–unemployed	0.34 (−1.52)	0.95 (−1.33)	−0.11 (−1.73)	−0.69 (−1.49)	0.03 (−1.59)
Unskilled–retired	−1.19 (−1.18)	−1.04 (−1.06)	−1.35 (−1.3)	−1.36 (−1.11)	−1.52 (−1.12)
Unskilled–other inactive	0.49 (−1.17)	0.39 (−1.05)	−0.84 (−1.42)	−0.37 (−1.21)	−1.44 (−1.21)
**Typology of smartphone use (ref: social media and entertainment)**					
Broad non-social-media users			−0.29 (−0.9)	−0.06 (−0.75)	0.15 (−0.81)
Basic general users			−0.4 (−0.79)	−0.68 (−0.65)	−0.34 (−0.71)
Camera users			−3.83 *** (−1.13)	−3.99 *** (−0.95)	−4.37 *** (−0.99)
Advanced users			1.75 * (−0.7)	1.29 * (−0.57)	1.54 * (−0.63)
**Cross-level interaction terms**					
Skilled–unemployed * User cannot interrupt the data collection	−0.5 (−0.54)				
Skilled–retired * User cannot interrupt the data collection	−1.13 ** (−0.43)				
Skilled–other inactive * User cannot interrupt the data collection	−0.21 (−0.37)				
Unskilled–employed * User cannot interrupt the data collection	1.09 ** (−0.36)				
Unskilled–unemployed * User cannot interrupt the data collection	1.26 (−0.83)				
Unskilled–retired * User cannot interrupt the data collection	0.38 (−0.62)				
Unskilled–other inactive * User cannot interrupt the data collection	−0.11 (−0.61)				
Skilled–unemployed * User can interrupt the data collection and has control over their data	−0.73 (−0.59)				
Skilled–retired * User can interrupt the data collection and has control over their data	−1.29 ** (−0.44)				
Skilled–other inactive * User can interrupt the data collection and has control over their data	−0.02 (−0.41)				
Unskilled–employed * User can interrupt the data collection and has control over their data	0.56 (−0.4)				
Unskilled–unemployed * User can interrupt the data collection and has control over their data	0.69 (−0.98)				
Unskilled–retired * User can interrupt the data collection and has control over their data	0.27 (−0.64)				
Unskilled–other inactive * User can interrupt the data collection and has control over their data	0.84 (−0.7)				
Skilled–unemployed * After the end of the research		0.98 (−0.51)			
Skilled–retired * After the end of the research		−0.18 (−0.37)			
Skilled–other inactive * After the end of the research		−0.16 (−0.33)			
Unskilled–employed * After the end of the research		−0.08 (−0.33)			
Unskilled–unemployed * After the end of the research		0.3 (−0.84)			
Unskilled–retired * After the end of the research		0.13 (−0.54)			
Unskilled–other inactive * After the end of the research		0.63 (−0.53)			
Skilled–unemployed * Both after downloading the app and at the end of the research		−0.05 (−0.52)			
Skilled–retired * Both after downloading the app and at the end of the research		0.3002			
Skilled–other inactive * Both after downloading the app and at the end of the research		−0.26 (−0.36)			
Unskilled–employed * Both after downloading the app and at the end of the research		0.13 (−0.35)			
Unskilled–unemployed * Both after downloading the app and at the end of the research		0.02 (−0.74)			
Unskilled–retired * Both after downloading the app and at the end of the research		0.43 (−0.63)			
Unskilled–other inactive * Both after downloading the app and at the end of the research		0.55 (−0.59)			
Scientific research institute * Broad non-social-media users			0.85 * (−0.4)		
Decision-maker * Broad non-social-media users			0.09 (−0.45)		
Scientific research institute * Basic general users			0.33 (−0.36)		
Decision-maker * Basic general users			−0.16 (−0.41)		
Scientific research institute * Camera users			−0.08 (−0.61)		
Decision-maker * Camera users			−0.78 (−0.69)		
Scientific research institute * Advanced users			0.02 (−0.35)		
Decision-maker * Advanced users			−0.06 (−0.38)		
Broad non-social-media users * After the end of the research				0.22 (−0.39)	
Basic general users * After the end of the research				0.35 (−0.34)	
Camera users * After the end of the research				0.04 (−0.6)	
Advanced users * After the end of the research				0.43 (−0.32)	
Broad non-social-media users * Both after downloading the app and at the end of the research				−0.52 (−0.47)	
Basic general users * Both after downloading the app and at the end of the research				−0.02 (−0.44)	
Camera users * Both after downloading the app and at the end of the research				−0.83 (−0.71)	
Advanced users * Both after downloading the app and at the end of the research				1.05 * (−0.41)	
Broad non-social-media users * User cannot interrupt the data collection					−0.42 (−0.45)
Basic general users * User cannot interrupt the data collection					−0.23 (−0.39)
Camera users * User cannot interrupt the data collection					0.73 (−0.69)
Advanced users * User cannot interrupt the data collection					0.48 (−0.37)
Broad non-social-media users * User can interrupt the data collection and has control over their data					−0.04 (−0.59)
Basic general users * User can interrupt the data collection and has control over their data					−0.44 (−0.53)
Camera users * User can interrupt the data collection and has control over their data					−1.38 (−0.83)
Advanced users * User can interrupt the data collection and has control over their data					1.10 * (−0.5)
AIC	6720.69	6792.82	5236.79	5253.7	5198.44
BIC	7052.36	7124.49	5543.22	5560.14	5504.88
Observations	10,000	10,000	7820	7820	7820
Groups (respondents)	1000	1000	782	782	782

Items of the Smartphone User Typology

(1) The 15 different activities for which one can use a smartphone.

**Table A2:** Do you use your smartphone to do the following (1—yes, 0—no).

	Yes	No	DK
Browsing websites	1	2	8
2. Writing/reading emails	1	2	8
3. Taking photos, videos	1	2	8
4. View content on social networking sites (e.g., texts, pictures, videos on Facebook, Instagram, Twitter, etc.)	1	2	8
5. Posting content to social networking sites (e.g., sharing texts, pictures, videos on Facebook, Instagram, Twitter)	1	2	8
6. Online shopping (e.g., tickets, books, clothes, technical goods)	1	2	8
7. Online banking (e.g., balance enquiries, transfers)	1	2	8
8. Install new apps (e.g., via Google Play or the App Store)	1	2	8
9. Use apps that use your device’s location (e.g., Google Maps, Foursquare)	1	2	8
10. Connect devices to your device via Bluetooth (e.g., smartwatch, pedometer)	1	2	8
11. Game	1	2	8
12. Listening to music, watching videos	1	2	8
13. Recording of training data (e.g., during a run, number of steps per day, etc.)	1	2	8
14. Read, edit files related to work, learning	1	2	8
15. Voice assistant services (Google Assistant, Apple Siri, Amazon Alexa, etc.)	1	2	8

(2) The average self-estimated daily duration of smartphone screen time.

Overall, how would you rate your knowledge and skills in using a smartphone? Please rate on a scale of 1 to 5.

Very Bad				Excellent	DK	Refusal
1	2	3	4	5	8	9

(3) Self-reported smartphone use skills.

On an average day, approximately how much time do you use your smartphone for work or personal use? Please specify in hours and minutes.

… Hours … Minutes
